# [^18^F]FDG-PET/CT atypical response patterns to immunotherapy in non-small cell lung cancer patients: long term prognosis assessment and clinical management proposal

**DOI:** 10.1007/s00259-024-06794-8

**Published:** 2024-06-19

**Authors:** Mathilde Masse, David Chardin, Pierre Tricarico, Victoria Ferrari, Nicolas Martin, Josiane Otto, Jacques Darcourt, Victor Comte, Olivier Humbert

**Affiliations:** 1 Centre Antoine Lacassagne, Nuclear Medicine Department, 33 Avenue de Valombrose, 06100 Nice, France; 2 Université Côte D’Azur, CNRS, Inserm, iBV, Nice, France; 3 Centre Antoine Lacassagne, Oncology Department, 33 Avenue de Valombrose, 06100 Nice, France; 4 TIRO-UMR E 4320, UCA/CEA, 28 Avenue de Valombrose, 06100 Nice, France

**Keywords:** Immunotherapy, PET/CT, Atypical response, Pseudoprogression, Dissociated response

## Abstract

**Aim:**

To determine the long-term prognosis of immune-related response profiles (pseudoprogression and dissociated response), not covered by conventional PERCIST criteria, in patients with non-small-cell lung cancer (NSCLC) treated with immune checkpoint inhibitors (ICPIs).

**Methods:**

109 patients were prospectively included and underwent [^18^F]FDG-PET/CT at baseline, after 7 weeks (PET_interim_1), and 3 months (PET_interim_2) of treatment. On PET_interim_1, tumor response was assessed using standard PERCIST criteria. In the event of PERCIST progression at this time-point, the study design provided for continued immunotherapy for 6 more weeks. Additional response patterns were then considered on PET_interim_2: pseudo-progression (PsPD, subsequent metabolic response); dissociated response (DR, coexistence of responding and non-responding lesions), and confirmed progressive metabolic disease (cPMD, subsequent homogeneous progression of lesions). Patients were followed up for at least 12 months.

**Results:**

Median follow-up was 21 months. At PET_interim_1, PERCIST progression was observed in 60% (66/109) of patients and ICPI was continued in 59/66. At the subsequent PET_interim_2, 14% of patients showed PsPD, 11% DR, 35% cPMD, and 28% had a sustained metabolic response. Median overall survival (OS) and progression-free-survival (PFS) did not differ between PsPD and DR (27 vs 29 months, p = 1.0; 17 vs 12 months, p = 0.2, respectively). The OS and PFS of PsPD/DR patients were significantly better than those with cPMD (29 vs 9 months, p < 0.02; 16 vs 2 months, p < 0.001), but worse than those with sustained metabolic response (p < 0.001). This 3-group prognostic stratification enabled better identification of true progressors, outperforming the prognostic value of standard PERCIST criteria (p = 0.03).

**Conclusion:**

[^18^F]FDG-PET/CT enables early assessment of response to immunotherapy. The new wsPERCIST (“wait and see”) PET criteria proposed, comprising immune-related atypical response patterns, can refine conventional prognostic stratification based on PERCIST criteria.

**Trial registration:**

HDH F20230309081206. Registered 20 April 2023. Retrospectively registered.

**Supplementary Information:**

The online version contains supplementary material available at 10.1007/s00259-024-06794-8.

## Introduction

Non-small-cell lung cancer (NSCLC) is one of the leading causes of cancer mortality worldwide [1]. In the metastatic setting of NSCLC, significant progress has been made in recent years with the development of immune checkpoint inhibitors (ICPIs), allowing the reactivation of anti-tumor immunity. ICPIs have revolutionized the management of metastatic lung cancer by significantly increasing overall and progression-free survivals compared to conventional chemotherapies[2–4], even achieving durable responses in some cases [5]. However, only about half of patients will derive a clinical benefit from this treatment [2–4, 6]. It is therefore of great importance to identify biomarkers that can predict treatment response or provide early assessment of treatment efficacy. Fluorine-18-fluorodeoxyglucose ([^18^F]FDG) positron emission tomography (PET)/ computed tomography (CT) is an imaging modality increasingly used to monitor treatment response in patients with metastatic NSCLC. The performance of PET/CT appears promising in the early differentiation of immunotherapy responders and non-responders [7, 8]. However, as for the RECIST 1.1 criteria [9], the PERCIST criteria [10] used to assess metabolic response to conventional treatments are not suitable for assessing response to immunotherapy due to its biological mechanism of action. By stimulating the patient's immune system to attack tumor cells, ICPIs induce immune cell infiltration of tumor cells, sometimes leading to a local inflammatory process and a transient increase in lesion size and/or [^18^F]FDG uptake, or even the appearance of new lesions. This initial lesion growth is followed by a delayed response and is termed “pseudoprogression” [11, 12]. The use of PERCIST criteria in this situation leads to an overestimation of ICPI failure and to discontinuation of treatment in patients for whom ICPI was actually effective. To account for this, modified PERCIST criteria have been proposed in sparse retrospective studies [13–15] but none are currently recommended in clinical practice due to lack of prospective validation [16, 17]. Currently, guidelines suggest that in case of doubt between progression or pseudoprogression and clinical stability, a confirmatory follow-up [^18^F]FDG-PET/CT should be performed 4–8 weeks later. This allows for atypical response patterns that have been described in immunotherapy, such as pseudoprogression, for which the prognosis appears to be different from that of classical progression [18]. Dissociated response is a less-known atypical pattern also described under ICPIs, defined as the coexistence of responding and non-responding lesions within the same patient [19]. In the face of such a response profile, the question arises whether to continue or discontinue treatment. To date, there has been no large-scale prospective study on the incidence of dissociated response, the criteria for its definition, and its long-term prognostic impact [20]. The aim of this study was to describe the long-term prognosis of atypical response patterns in the setting of immunotherapy (i.e. pseudoprogression and dissociated response) as compared to prognosis of conventional PERCIST response profile. On the basis of the results, we aimed to formulate clinical recommendations for NSCLC patients, treated with ICPIs and follow-up with PET/CT.

## Methods

### Patient inclusion criteria

This ancillary study is predicated on a combination of two current-care studies conducted at our institution. Both studies were prospective, observational, uncontrolled, and non-randomized, investigating the value of standard [^18^F]FDG-PET/CT in monitoring NSCLC response to immunotherapy:

- The first study prospectively enrolled 99 patients from February 2017 to June 2022 (FDG ECMI n°ID-RCB: 2018-A02116-49).
- The second study prospectively enrolled 43 patients from April 2019 to June 2022 (FDG IMMUN, NCT03584334).

Characterizing the prognostic value of atypical response patterns on PET/CT was a secondary objective of both studies. Inclusion criteria for both studies were identical: (1) pathologically proven stage IIIB or IV NSCLC; (2) an indication to start ICPI monotherapy as a first or later line of treatment; (3) Eastern Cooperative Oncology Group (ECOG) performance status of 0 to 2; (4) age of at least 18 years; (5) no prior immunotherapy. Exclusion criteria were: (1) clinical or biological contraindication for ICPIs; (2) vulnerable patients as defined in Article L1121-5 to L1121-8 of the French Public Health Code; (3) high glycemia (≥ 11 mmol/L) at baseline [^18^F]FDG-PET/CT exam; (4) a delay greater than 3 months between baseline PET and initiation of immunotherapy; (5) refusal of written consent; (6) no measurable lesion by PERCIST V1.0; (7) evidence of concomitant progressive cancer; (8) histologic subtype other than adenocarcinoma, squamous cell carcinoma or undifferentiated carcinoma.

Patients received one of the following three ICPI drugs: pembrolizumab 2mg/kg or 200mg every 3 weeks, nivolumab at a standard dose of 240mg/2 weeks, or atezolizumab at a standard dose of 1200mg/3 weeks.

### PET protocol

All patients underwent a [^18^F]FDG-PET/CT within 12 weeks prior to the initiation of treatment (PET_baseline_), 7 weeks after the start of treatment (PET_interim_1), and 3 months after the start of treatment (PET_interim_2). [^18^F]FDG-PET/CT was performed using two different PET/CT imaging systems: Biograph mCT PET/CT from February 2017 to September 2019 and Biograph Vision 600 PET/CT from September 2019 to June 2022 (Siemens Healthcare, Erlangen, Germany). Both had EARL accreditation for FDG-PET/CT tumor imaging. The patients were asked to fast for at least 6 h before the intravenous injection of 3 MBq/kg (Biograph mCT) or 2.5 MBq/kg (Biograph Vision 600) of [^18^F]FDG. A low-dose attenuation CT acquisition (80 kV, 50 mA, 5 mm slice thickness) was performed 60 ± 5 min after the administration of [^18^F]FDG, followed by an inspiratory chest-restricted diagnostic CT (auto-kV, auto-mA, 1-mm-slice thickness). Lastly, a diagnostic CT acquisition was done from the skull to mid-thigh (auto-kV, auto-mA, 1-mm-slice thickness) after a venous injection of iodinated contrast agent in the absence of allergy or renal impairment. Baseline and follow-up exams were performed using the same imaging system and acquisition parameters. The patient weight-normalized SUVpeak was calculated on a region of 10mm diameter centered on the most avid focus. The target lesions were defined as the five most avid lesions on each PET exam, as there may be differences between the different exams. Hyper-avid foci due to inflammation induced by immunotherapy were excluded from the analysis. Image were analyzed by two blinded primary readers, and a third blinded reader resolved disagreements.

### PET_interim_1: early assessment after 7 weeks of treatment

#### PERCIST criteria

Metabolic response was first assessed according to the PERCIST criteria [10] (Table S1). Briefly, a complete metabolic response (CMR) was defined as the disappearance of target lesion uptake with no new lesion, a partial metabolic response (PMR) was defined as a decrease in the sum of the SUVpeak of the target lesions by more than 30% with no new lesion, a stable metabolic disease (SMD) was defined as the absence of criteria to define progression, partial or complete metabolic response. All of these responses define a group of patients termed “metabolic responders” (MR). Finally, progressive metabolic disease (PMD) was defined as new lesions and/or progression of target lesions with an increase in the sum of SUVpeak by more than 30%.

Due to the experimental nature of PET_interim_1 and the known specific immune-related response pattern, the study protocol recommended continuing ICPI treatment beyond a first PERCIST progression, except in the case of severe clinical deterioration, in accordance with the multi-disciplinary tumor board decision. Patient safety was monitored throughout, and treatment could be halted if there was any clinical worsening or toxicity.

### PET_interim_2: late assessment after 3 months of treatment

#### PERCIST criteria

The metabolic response at 3 months was again evaluated according to the PERCIST criteria (Table S1).

#### Atypical evolutive patterns

For the specific group of patients with a first PERCIST PMD at 7 weeks (on PET_interim_1), the 3-month metabolic response (on PET_interim_2) was also defined adding 3 new atypical patterns:

- *Pseudoprogression (PsPD)*: in case of CMR, PMR, or SMD at 3 months.
- *Dissociated response (DR)*: in case of heterogeneous metabolic evolution of lesions, i.e. in case of coexistence of responding and non-responding lesions on the same PET scan.
- *Confirmed PMD (cPMD)*: in case of homogeneous metabolic progression of all lesions.

### Follow-up and outcomes

Patients were followed for at least 12 months, with regular clinical assessments and standard imaging tests (i.e. CT or PET/CT every 3 months, or sooner if clinical progression was suspected ± brain MRI if a brain localization was clinically suspected). The delay between the initiation of treatment and the decision to stop it, as well as the reason why (tumor progression, toxicity, therapeutic break, patient’s refusal to continue the treatment) was recorded.

The primary endpoint was overall survival (OS) defined as the time from initial immunotherapy to death from any cause.

Progression-free survival (PFS) was a secondary endpoint of the study and defined as the time from the initiation of ICPI to confirmed tumor progression or death. Tumor progression, leading to discontinuation of treatment, had to be confirmed by a multi-disciplinary tumor board, comparing the patient’s clinical status and a detailed analysis of images. For PFS, we did not take into account the time of first evidence of tumor progression on PET/CT due to pseudo-progression or dissociated response patterns.

### Statistical analyses

Data management was performed using the Ennov Clinical Software Suite. Quantitative variables are indicated by their mean (standard deviation) and qualitative values are designated by their absolute value followed by the percentage. We used Kaplan–Meier’s method to estimate survival outcomes, which were compared using the log-rank test between the groups. Patients were censored at the end of follow-up or the date of the latest news. Univariate and multivariate analyses were performed using the Cox proportional hazards regression. A p-value < 0,05 was used to attest statistical significance. Statistical analyses were performed using R (version 2022.12.0 + 353).

## Results

### Patient characteristics

Of the 142 eligible patients, 124 were finally included. Others were excluded from the study because they had a non-eligible histological type (N = 3), they were lost to follow-up before one year (N = 3), they had a concomitent progressive cancer (N = 1), immunotherapy was finally non initiated after patient inclusion (N = 4), presented one or more of target lesions that had received radiotherapy (N = 2), poor image quality (N = 1), economic reasons (N = 1), delay greater than 3 months between PET baseline and ICPI introduction or 9 weeks between introduction of ICPIs and PET_interim_1 (N = 3). Of the patients included, some did not undergo the PET_interim_1 exam because of early discontinuation of ICPI due to clinical deterioration (N = 12) or treatment toxicity (N = 3) (Supplemental Fig. S1). Consequently, 109 patients were finally analyzed.

The mean patient age was 64.5 years, most patients were men (64%), current or former smokers (85%), had an adenocarcinoma histology (82%) and received pembrolizumab (56%) or nivolumab (40%) (Table 1). Mean time between PET_baseline_ and initiation of immunotherapy was 14.7 days [range 0–74; SD 15.7]. Median duration of immunotherapy was 8 months [range 1–60]. Median patient follow-up was 21 months [Q1-Q3 9–38]. By the end of the study, 73 patients had died and 4 were lost to follow-up. 70 patients progressed during follow-up and 71% received a subsequent line of treatment. Median OS was 23 months [IC95% 18–38] and median PFS was 13 months [IC95% 7–23].

**Table 1:** Patient characteristics

		All (N = 109)	
		n	%
Age in years, mean (SD)		64.5 (9.5)	
Gender	Male	70	64
	Female	39	36
Smoking status	Never	17	16
	Former smokers	50	46
	Current smokers	42	39
Immunotherapy	Pembrolizumab	61	56
	Nivolumab	44	40
	Atezolizumab	4	4
Histology	Adenocarcinoma	89	82
	Squamous cell carcinoma	18	17
	Undifferentiated	2	2
PDL1 status	< 50%	42	39
	> 50%	47	43
	Unknown	20	18
Anterior surgery	Yes	24	22
	No	85	78
Anterior radiotherapy	Yes	43	39
	No	66	61
Anterior chemotherapy	Yes	79	72
	No	30	28
Number of prior chemotherapy	0	30	28
	1	42	39
	2	24	22
	3	13	12

### PET_interim_1: early assessment after 7 weeks of treatment

Mean time between introduction of immunotherapy and PET_interim_1 was 49 days (range 34–64; SD 5.6).

#### PERCIST criteria

39,5% (43/109) of patients were metabolic responders (MR) according to PERCIST criteria:

- 10,1% (11/109) of patients had a complete metabolic response (CMR). All were alive at the end of the study. Only one progressed 18 months after the introduction of treatment.
- 20,2% (22/109) of patients had a partial metabolic response (PMR) and the median OS and PFS were not reached.
- 9,2% (10/109) of patients had stable metabolic disease (SMD), the median OS was 21.5 months [95%CI 8-not reached], and the median PFS was 13 months [95%CI 6-not reached].

In contrast, 60.5% (66/109) of patients had a progressive metabolic disease (PMD) according to PERCIST criteria, the median OS was 15 months [95%CI 11–23], and the median PFS was 5 months [95%CI 3–8] (Table 2).

**Table 2:** Response according to PERCIST criteria on PET_interim_1 and PET_interim_2

	Number		Median OS* (95%IC)	Median PFS* (95%IC)	p-value
	n	%			
PERCIST criteria on PET_interim_1					
MR	43	39.5			< 0 .001
CMR	11	10.1	NR	NR	
PMR	22	20.2	NR	NR	
SMD	10	9.2	21.5 (8-NR)	13 (6-NR)	
PMD	66	60.5	15 (11–23)	5 (3–8)	
PERCIST criteria on PET_interim_2					
CMR	15	14	NR	NR	< 0.001
PMR	21	19	NR	NR	
SMD	9	8	49 (23-NR)	47 (29-NR)	
PMD	63	58	15 (10–20)	3 (3–9)	

^*^ In months

*CMR*, Complete Metabolic Response; *PMR,* Partial Metabolic Response; *SMD*, Stable Metabolic Disease; *PMD*, Progressive Metabolic Disease; *MR*, Metabolic Response; *NR*, Not Reached

There was a significant difference in overall and progression-free survival curves between the different PERCIST sub-groups on PET_interim_1 (p < 0.001) (Supplemental Fig. S2).

### PET_interim_2: late assessment after 3 months of treatment

Mean time between introduction of immunotherapy and PET_interim_2 was 13.8 weeks (range 11–17; SD 1.9).

Among the 109 patients that benefited from the PET_interim_1, 23 did not benefit from the PET_interim_2 because of:

- Significant clinical worsening before PET_interim_2 leading to confirmation of tumor progression and early treatment stop decided by the multi-disciplinary tumor board (N = 17). These patients were kept for analysis as patients with tumor progression at the time of PET_interim_2.
- Complete metabolic response on PET_interim_1 (N = 5) and a subsequent PET _interim_2 still showing a CMR but performed more than 3 months after treatment initiation: they were classified as CMR on PET_interim_2.
- PET exam cancellation without reason (N = 1): this patient was excluded from the analysis.

**Fig. 1:**
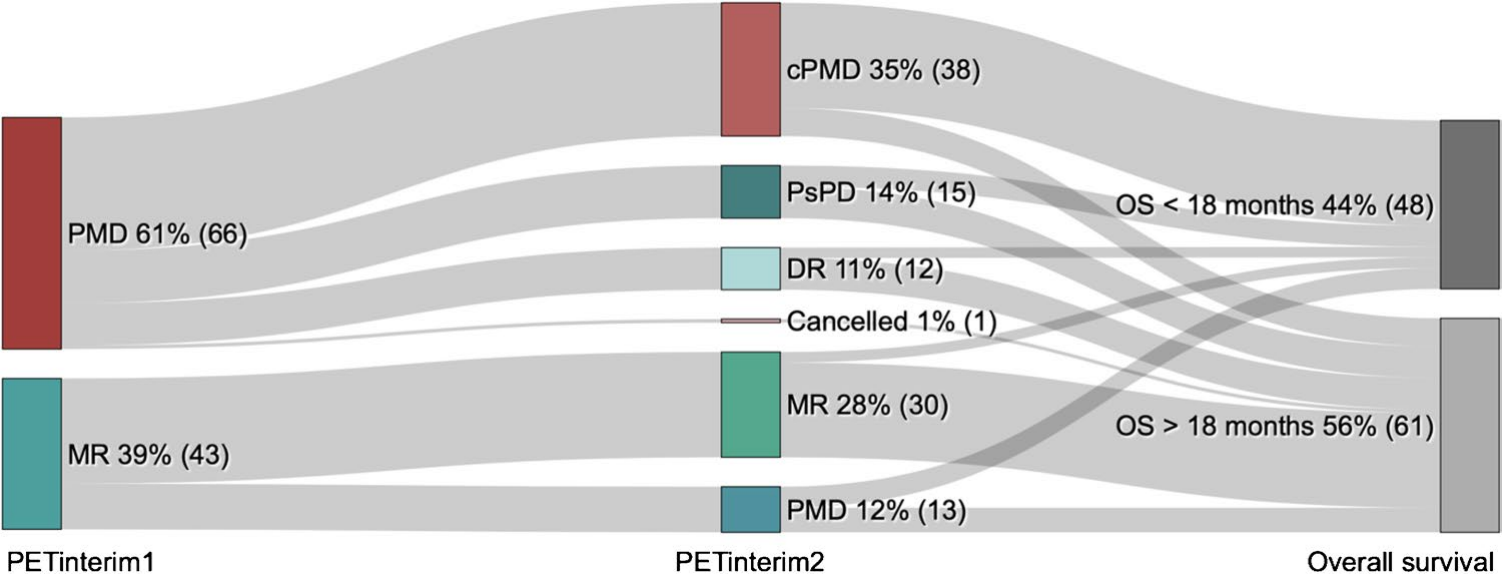
Sankey Diagram on patient outcomes between PETinterim1-PETinterim2 and survival

The PET_interim_2s of 108 patients were therefore analyzed (Fig. 1).

PMD: Progressive Metabolic Disease, MR: Metabolic Response (i.e. sustained CMR, PMD or SMD), DR: Dissociated Response, PsPD: Pseudoprogression, cPMD: confirmed Progressive Metabolic Disease, OS: Overall Survival.

#### PERCIST criteria

According to the PERCIST criteria comparing PET_interim_2 to baseline:

- 15 patients (14%) had a CMR: the median OS and PFS were not reached.
- 21 patients (19%) had a PMR: the median OS and PFS were not reached.
- 9 patients (8%) had SMD: the median OS was 49 months [95%CI 23-not reached] while the median PFS was 47 months [95%CI 29-not reached].
- 63 patients (58%) had PMD: the median OS was 15 months [95%CI 10–20] while the median PFS was 3 months [95%CI 3–6] (Table 2).

There is a significant difference in overall and progression-free survival between the different groups (p < 0.001) (Supplemental Fig. S3).

#### Atypical evolutive patterns

Among the 66 patients with a first PERCIST progression on PET_interim_1, 65 were analyzed on PET_interim_2:

- 15/65 (23%) subsequently showed a complete, partial, or stable metabolic response, a posteriori indicating an initial pseudoprogression (PsPD), with a median OS of 27 months [95%CI 13-unreached] and median PFS of 17 months [95%CI 8-unreached].
- 12/65 (18.5%) showed a dissociated response (DR) with a median OS of 29 months [95%CI 19-unreached] and a median PFS of 12 months [95%CI 9-unreached].

38/65 (58.5%) showed a cPMD (confirmed Progressive Metabolic Disease = subsequent homogeneous progression of lesions) with a median OS of 9 months [95%CI 6–15] and median PFS of 2 months [95%CI 2–3].

OS and PFS were not statistically different between patients with pseudoprogression and those with dissociated response (p = 1, p = 0.2, respectively). They were therefore grouped together for the following statistical analyses.

**Fig. 2:**
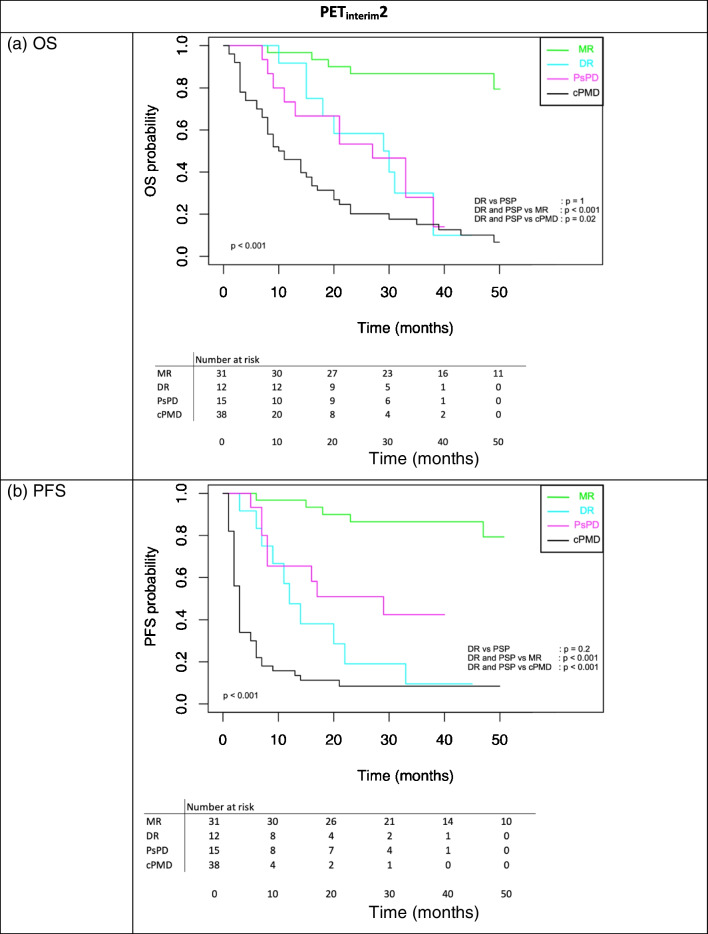
Kaplan–Meier survival curves showing overall survival (OS) (**a**) and progression free survival (PFS) (**b**) according to tumor response on PET_interim_2 including atypical response. MR: Metabolic Response (i.e. sustained CMR, PMD or SMD on PET_interim_1 and PET_interim_2), DR: Dissociated Response, PsPD: Pseudoprogression, cPMD: confirmed Progressive Metabolic Disease

Statistical comparison of patient survival by metabolic response category showed (Fig. 2a and 2b) that patients with a PsPD or cDR at 3 months had:

- significantly better median OS and median PFS than patients with cPMD (29 vs 9 months, p < 0.02 and 16 vs 2 months, p < 0.001, respectively).
- significantly worse median OS and median PFS than patients who maintained a metabolic response on the 2 successive PET exams (29 months vs not reached, p < 0.001 and 16 months versus not reached, p < 0.001, respectively).

**Fig. 3:**
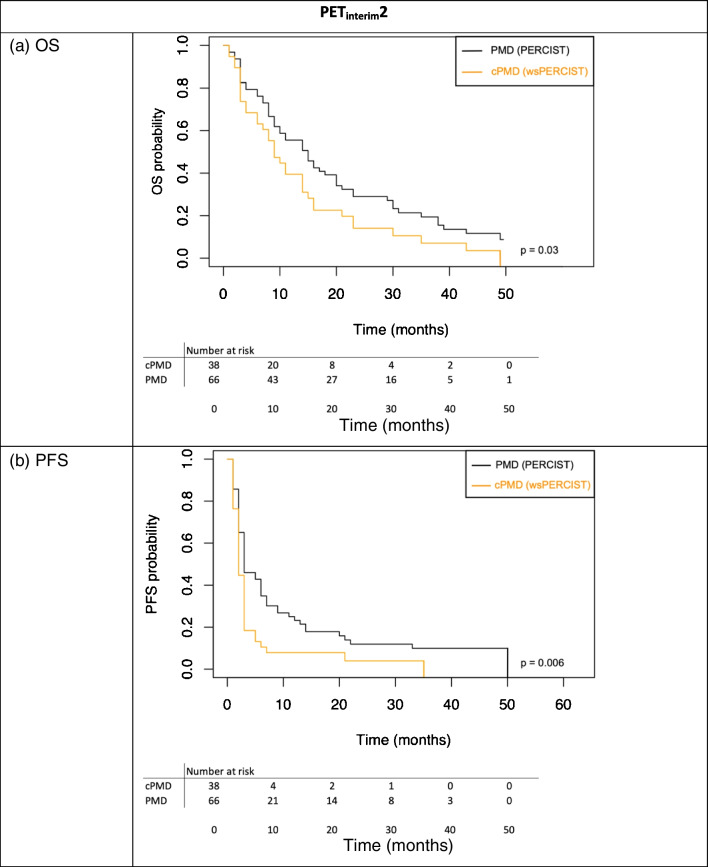
Kaplan–Meier survival curves showing overall survival (OS) (**a**) and progression free survival (PFS) (**b**) in patients with PMD according to PERCIST and with cPMD on PET_interim_2. PMD: Progressive Metabolic Disease, cPMD: confirmed Progressive Metabolic Disease

Furthermore, when comparing on PET_interim_2 the outcome of patients with PERCIST PMD with those with cPMD (thus eliminating pseudoprogression and dissociated response), patients with cPMD had significantly worse median OS and median PFS than patients with a standard PERCIST PMD (9 vs 15 months, p = 0.03 and 2 vs 3 months, p = 0.006, respectively) (Fig. 3a and 3b).

Univariate and multivariate analysis including the clinical parameters did not reveal any clinical confounding factors in terms of OS and PFS (Fig. S4).

## Discussion

### Atypical response in the setting of ICPIs

Early and accurate assessment of treatment efficacy is critical for patients undergoing cancer treatment. Imaging-based evaluation appears to be a promising biomarker for assessing the efficacy of immunotherapy [21, 22]. However, the current imaging criteria used for evaluating treatment efficacy, either RECIST 1.1 for CT [9] or PERCIST 1.0 for PET/CT [10], were established primarily for chemotherapy and targeted therapies and are not fully suitable for immunotherapy. Indeed, the mechanism of action of immunotherapy differs from that of chemotherapy and targeted therapies, as it involves the activation of the immune system to fight cancer cells and may induce an inflammatory process [23]. This leads to distinct response patterns that are not captured by current conventional imaging criteria and are therefore often referred to as progression as they increase tumor size or tumor burden. As well described by Hodi et al. [24], in a study conducted on patients with metastatic melanoma treated with pembrolizumab, with CT scan monitoring, conventional RECIST criteria led to progression misclassification of 15% of patients. In our study, using PERCIST criteria, 60% of patients were classified as having metabolic progression 7 weeks after treatment initiation, while only 35% had confirmed metabolic progression on the control [^18^F]FDG-PET/CT performed 6 weeks later, suggesting that almost 25% of patients were misclassified using PERCIST criteria on the first PET exam. These patients actually exhibited an atypical response profile to immunotherapy. Conventional criteria may therefore lead to overdiagnosis of progression, resulting in premature discontinuation of ICPI in patients who could potentially have benefited from it [25, 26].

The most common atypical response profile described with immunotherapy is pseudoprogression (Fig. S5). It was first described in patients treated with anti-CTLA4 therapy for metastatic melanoma [27]. Pseudoprogression is defined as initial progression of both target and non-target lesions and/or appearance of new lesions followed by a response. Previous studies have shown that PsPD occurs in approximately 7% of patients monitored by CT scan [28, 29] and approximately 10% of patients monitored by [^18^F]FDG-PET/CT imaging [25]. In our observational study on prospectively gathered data, the prevalence of pseudoprogression was 14%.

**Fig. 4:**
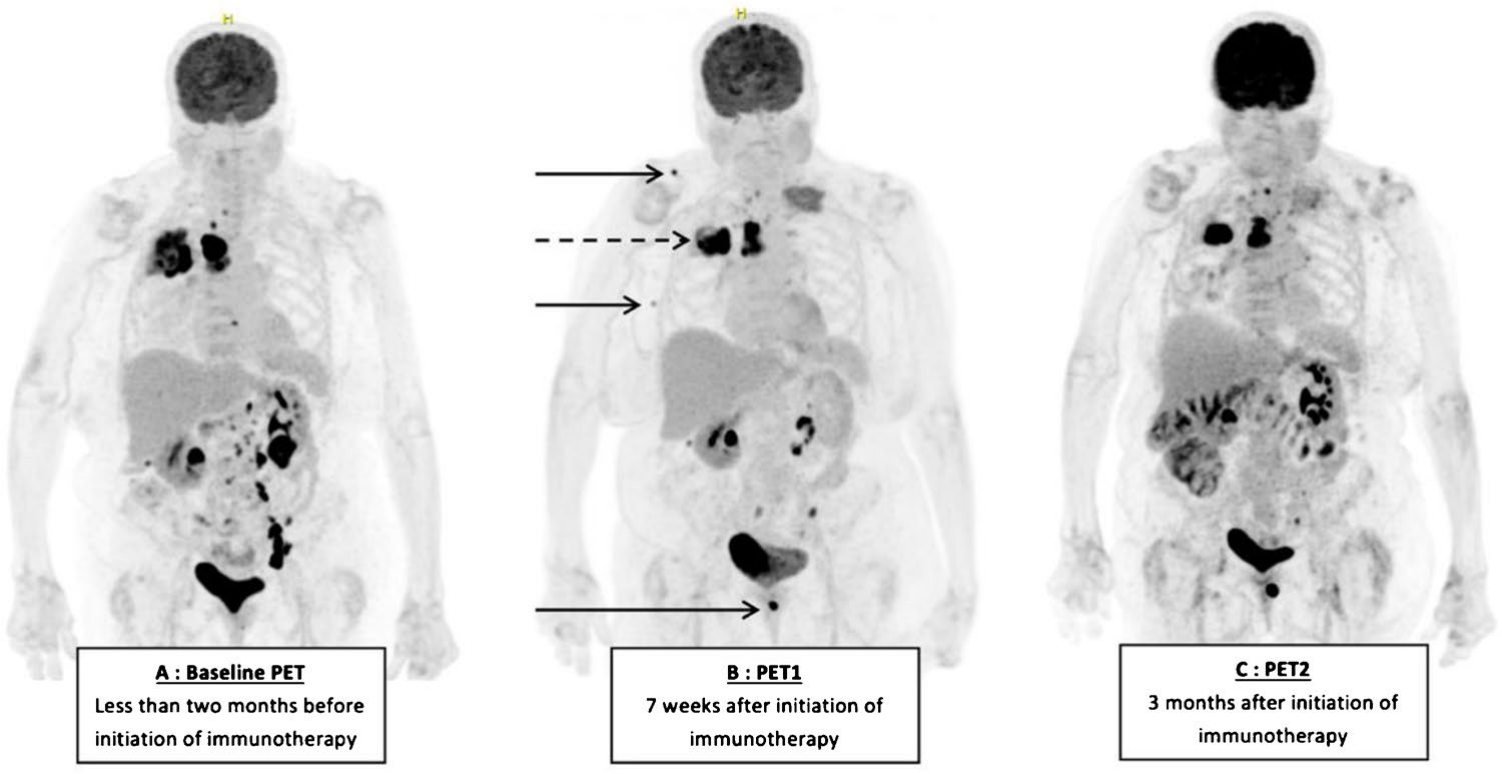
Progressive metabolic disease according to PERCIST 7 weeks after initiation of immunotherapy with progression of axillary and supraclavicular lymph nodes and skin lesion (continuous arrow) and partial response of retroperitoneal and mediastinal lymph nodes and pulmonary lesion (dotted arrow)(B). 3 months after initiation of immunotherapy, dissociated response was observed with all lesions shrinking except the skin (C)

The second atypical response profile is the dissociated response (Fig. 4). It is defined as progression of some lesions concomitant with shrinkage of others. This profile was first defined in 2010 in a study evaluating the response of metastatic breast cancer to systemic treatments (chemotherapy, targeted therapies, hormone therapy) using [^18^F]FDG-PET/CT imaging [30]. Although initial studies have suggested a specific prognosis for this type of response, it has consistently been considered an unfavorable prognostic pattern and therefore been included in the "progressive disease" category of RECIST 1.1 or PERCIST 1.0. Actually, we believe that the dissociated response combines several local pseudoprogression phenomena. On CT scan, its incidence under ICPIs is around 5–10% of patients, depending on the studies [28, 31–33]. In our study using [^18^F]FDG-PET/CT, the prevalence of dissociated response is much more common, occurring in 27% of patients after 7 weeks of treatment, and 11% after 3 months.

Our study reports a high rate of atypical responses compared to literature, which may raise the question of premature PET evaluation. Nevertheless, an early evaluation of NSCLC response, performed after 2–3 cycles of immunotherapy, is recommended by international bodies [34], mainly because of the aggressive nature of this cancer, which rules out later evaluation, especially at the beginning of treatment. Moreover, the higher incidence with [^18^F]FDG-PET/CT than with CT is due to the higher sensitivity of PET/CT and its ability to provide better lesion-by-lesion analysis, making it easier to detect these patterns compared to CT.

Although some retrospective studies have shown that patients with pseudoprogression [13, 24, 35, 36] or dissociated response [25, 31, 32, 37] have a better short-term outcome than patients with progression, no study has examined the long-term, specific prognosis of these atypical response patterns. Our long-term study using prospectively gathered data demonstrates that the prognosis of patients with pseudoprogression is similar to that of patients with a dissociated response occurring after an initial PERCIST progression. This prognosis is intermediate: much better than that of patients with confirmed progression (defined as 2 consecutive homogeneous progressions of lesions), but still worse than that of patients with sustained metabolic response after 3 months of treatment, on two successive PETs. A dissociated response, in contrast to pseudoprogression, can also be detected using early evaluation PET, 7 weeks after treatment initiation. Additional analyses have enabled us to show that a dissociated response at 7 weeks has a better prognosis than homogeneous progression (Fig. S6). However, we reckon that this type of response does not make it possible to predict further evolution since, in our study, approximately one third of patients will have subsequently confirmed progression, one third will have a second dissociated response, and one third will have a metabolic response on a PET performed 6 weeks later. Therefore, as with PsPD, the best time to consider the prognostic value of dissociated metastatic lung cancer response is on PET_interim_2, after an initial PERCIST progression.

### Physiopathology of response to ICPIs

The pseudoprogression phenomenon occurring in an atypical response (PsPD and DR) can be explained by tumor infiltration by immune cells, which are responsible for an increase in FDG uptake linked to the inflammatory reaction induced by the immune system [11]. We believe that this is probably systematic, or at least very frequent, in patients who respond to ICPI, as treatment efficacy is based on tumor immune infiltration.

However, the time after which this phenomenon is observed may vary according to the level of efficacy of the treatment:

- patients with a very good ICPI efficacy can experience pseudoprogression as early as the first week following the introduction of treatment, as suggested in a study by Anderson et al. [38]. This phenomenon was not observed in our study, which did not provide for such an early assessment; they were thus classified as metabolic responders since pseudoprogression had been missed.
- patients with a delayed treatment efficacy will experience pseudoprogression later (around a few weeks); they are the patients with an atypical response in our study. We assume that for these patients, immunotherapy effect is delayed and probably reduced, hence the intermediate prognosis observed in our study.

All these hypotheses need to be validated through dedicated studies.

### New criteria in the setting of IPIs

In order to take into account these atypical response profiles and to avoid misclassifying a patient as having metabolic progression, specific criteria have been proposed in the literature such as iPERCIST [13] or imPERCIST [14]. None of these criteria are currently recommended in guidelines for routine clinical use due to lack of validation in prospective studies with large patient cohorts. The current recommendation according to the EANM guidelines [16] is that in case of doubt between progression and pseudoprogression, clinicians should proceed with ongoing treatment and reassess with a follow-up [^18^F]FDG-PET/CT conducted 4 to 8 weeks after the initial scan, provided the patient's clinical condition permits. This is referred to as a “wait and see" strategy.

**Fig. 5:**
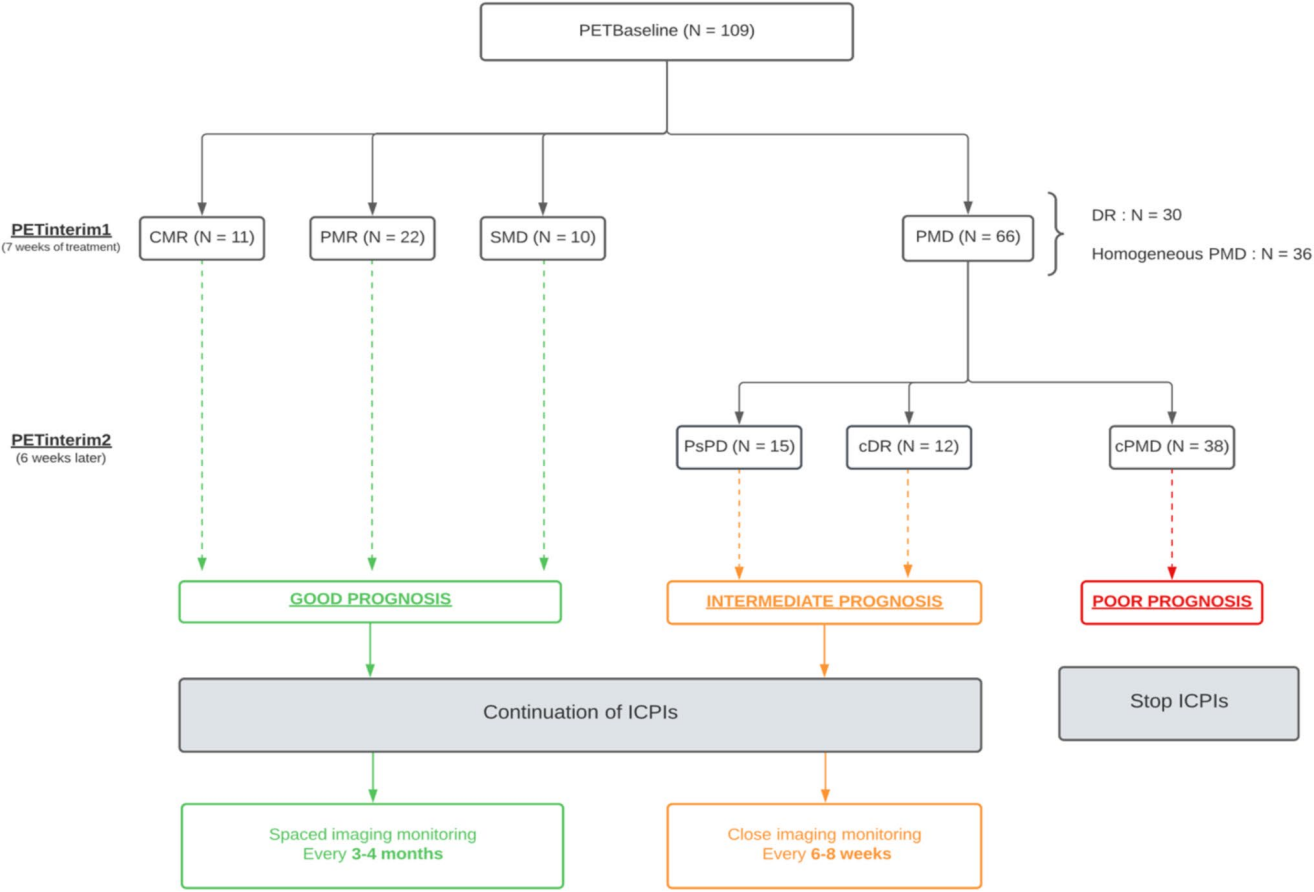
Decision-making algorithm to monitor response to ICPIs with [^18^F]FDG-PET/CT, using the wsPERCIST criteria

Based on this international recommendation [16] and the clinical results of the present observational study, we have proposed the new “wsPERCIST” criteria (Table 3). They build upon the existing PERCIST criteria but introduce two atypical response patterns, namely dissociated response and pseudoprogression. They suggest maintaining treatment following an initial PERCIST progression on PET_interim_1 and performing a second [^18^F]FDG-PET/CT 4–8 weeks later. At the time of PET_interim_2, three new patterns can then be identified: 1) Pseudoprogression (PsPD) in cases of subsequent CMR, PMR or SMD, 2) Dissociated Response (DR) in cases of concomitant responding and non-responding lesions, 3) Confirmed Progressive Metabolic Disease (cPMD) in cases of homogeneous progression of all lesions. In cases of cPMD, immunotherapy is considered ineffective and discontinuation of treatment should be considered by the multidisciplinary tumor board. However, in the case of an atypical response (PsPD and DR), immunotherapy should be continued, but imaging follow-up should be more frequent, every 6 to 8 weeks, due to their intermediate prognosis. Finally, in metabolic responders, imaging follow-up can be done as usual every 3 months, or even further apart, every 4 months, given their very good long-term prognosis (Fig. 5). These criteria allow a better stratification of patient prognosis by identifying 3 distinct prognostic groups. They outperform the standard PERCIST criteria by better identifying true progressors. This avoids premature discontinuation of immunotherapy in patients who would have benefited from it.

**Table 3:** Comparison of the definitions of PERCIST and wsPERCIST criteria for immunotherapy evaluation. *By comparing PET_*interim*_*2 to PET*_*interim*_*1*

	PERCIST	wsPERCIST	
Target lesions	Tumor SUL > 1.5 × SUL_mean(liver)_ + 2DS Maximum of 5 targets lesions, being the 5 hottest lesions, not necessarily the same on different follow-up exams		
Complete metabolic response (CMR)	Complete resolution of ^18^FDG uptake within target and nontarget lesions, indistinguishable from surrounding background blood-pool levels		
Partial metabolic response (PMR)	Reduction of minimum of 30% of the sum of the SUL_peak_ of target lesion(s) (minimum reduction of 0.8 units of SUL); No increase > 30% of SUL_peak_ of target or nontarget lesion(s); No new lesion		
Stable disease (SD)	Neither CMR, PMR or PMD		
Progressive metabolic disease (PMD)	> 30% increase of the sum of the SUL_peak_ of at target lesions or new ^18^FDG-avid lesion(s) that are typical of cancer and not related to the treatment effect or infection	In case of PMD, a second PET (PET_interim_2), 4–8 weeks after the previous one is needed*	
		Pseudoprogression (PsPD)	CMR, PMR or SMD according to PERCIST on PET_interim_2
		Dissociated response (DR)	PMD according to PERCIST on PET_interim_2 and on lesion by lesion analysis: • > 30% increase of SUL_peak_ of at least one target lesion or new ^18^FDG-avid lesion(s) • WITH decrease > 30% of SUL_peak_ of at least one target or nontarget lesion(s)
		Confirmed PMD (cPMD)	PMD according to PERCIST on PET_interim_2 and on lesion by lesion analysis: • > 30% increase of SUL_peak_ of at least one target lesion or new(s) ^18^FDG-avid lesions • WITHOUT decrease > 30% of SUL_peak_ of at least one target or nontarget lesion(s)

CMR: Complete Metabolic Response, PMR: Partial Metabolic Response, SMD: Stable Metabolic Disease, PMD: Progressive Metabolic Disease, PsPD: Pseudoprogression, cDR: confirmed Dissociated Response, cPMD: confirmed Progressive Metabolic Disease.

We also compared these new criteria (i.e. wsPERCIST) with those already proposed for immunotherapy (iPERCIST and imPERCIST [13, 14]), the main differences are as follows (Table S2):

- compared to iPERCIST, our criteria classify patients with dissociated response as responders, whereas iPERCIST would classify them as cPMD
- compared to imPERCIST, most patients with an atypical response according to our criteria are also classified as responders according to imPERCIST (20/27; 74%). Nevertheless, we believe it is important to distinguish between patients with a sustained metabolic response and those with an atypical response, as their prognosis is different; we recommend closer follow-up in the case of an atypical response.

### Strengths and limits of the study

Our study was conducted in a large cohort of patients with a median follow-up of over 2 years. Unlike other studies, it was specifically designed to evaluate tumor response based on PET/CT, and took account of atypical evolutive patterns. To our knowledge, no other study has evaluated the prognostic value of PET/CT imaging for immunotherapy monitoring with such long-term follow-up. Furthermore, the prospective design of the two studies used encouraged continuation of treatment in patients who showed progression on PET_interim_1 if their clinical condition permitted, which made it possible to assess the benefit of continuing treatment in patients with an atypical response after initial progression. This is crucial to properly observe the true rate of occurrence and prognostic significance of atypical response patterns to ICPIs.

Our study has limitations. First, our inclusion criteria allowed for a delay of 3 months between baseline PET scan and the start of treatment, which can be considered a fairly long interval. However, a delay of more than 6 weeks involved only 5 patients (out of 109) with various response profiles, which did not seem to affect the results. Also, it is a monocentric study with a limited number of patients exhibiting atypical responses (N = 27); it therefore appears important to externally confirm the prognostic relevance of the proposed wsPERCIST (“wait and see”) criteria in multicentric prospective studies. Additionally, we only included patients receiving immunotherapy alone. However, the combination of chemotherapy and ICPIs is now a standard for metastatic NSCLC and atypical responses have also been observed in these patients [39]. The incidence and prognosis of atypical response patterns, as well as the clinical value of the wsPERCIST criteria, will have to be studied in this specific therapeutic setting. Although we know that irAES provides additional prognostic information [40], we did not study this point, which warrants a dedicated study. Finally, future medico-economic studies will be needed to assess the cost/benefit ratio of PET/CT tumor response monitoring for both patients and the healthcare system.

In conclusion, long-term prognosis is associated with early response observed on PET/CT imaging and described according to PERCIST. However, these conventional criteria overestimate progression in 25% of patients, leading to potential discontinuation of ICPIs in patients who would have exhibited a clinical benefit. This large-scale, prospective, observational study enables us to propose novel criteria in line with the currently recommended "wait and see" strategy of the EANM guidelines [26], encompassing the definition of atypical responses (pseudoprogression and dissociated response). These atypical responses have a rather favorable prognosis and should not be conflated with disease progression.

## Supplementary Information

Below is the link to the electronic supplementary material.

Supplementary file1 (DOCX 1545 KB)

## Data Availability

The datasets generated during and/or analyzed during the current study are available from the corresponding author on reasonable request.
