# Supplementary material for: [18F]FDG-PET/CT atypical response patterns to immunotherapy in non-small cell lung cancer patients: long term prognosis assessment and clinical management proposal
Source: Eur J Nucl Med Mol Imaging. 2024 Jun 19;51(12):3696–708. doi: 10.1007/s00259-024-06794-8 (PMC11457717; doi:10.1007/s00259-024-06794-8)
Supplement: Supplementary file 1 — Supplementary file1 (DOCX 1545 KB) [file 259_2024_6794_MOESM1_ESM.docx]

**SUPPLEMENTARY DATA:**

| Progressive metabolic disease (PMD) | Increase > 30% of the sum of SULpeak of target lesions from baseline scan or  visible increase in extent of tumor uptake or  new avid lesions typical of cancer. |
| --- | --- |
| Stable metabolic disease (SMD) | Not CMR, PMR, or PMD. |
| Partial Metabolic Response (PMR) | Reduction > 30% of the sum of SULpeak of target lesions and absolute drop in SUL > 0.8 SUL units. |
| Complete Metabolic Response (CMR) | Complete resolution of FDG uptake within measurable target lesion (less than mean liver activity and indistinguishable from surrounding background blood-pool levels).  No new lesion typical of cancer. |

**Table S1** : PERCIST 1.0 criteria


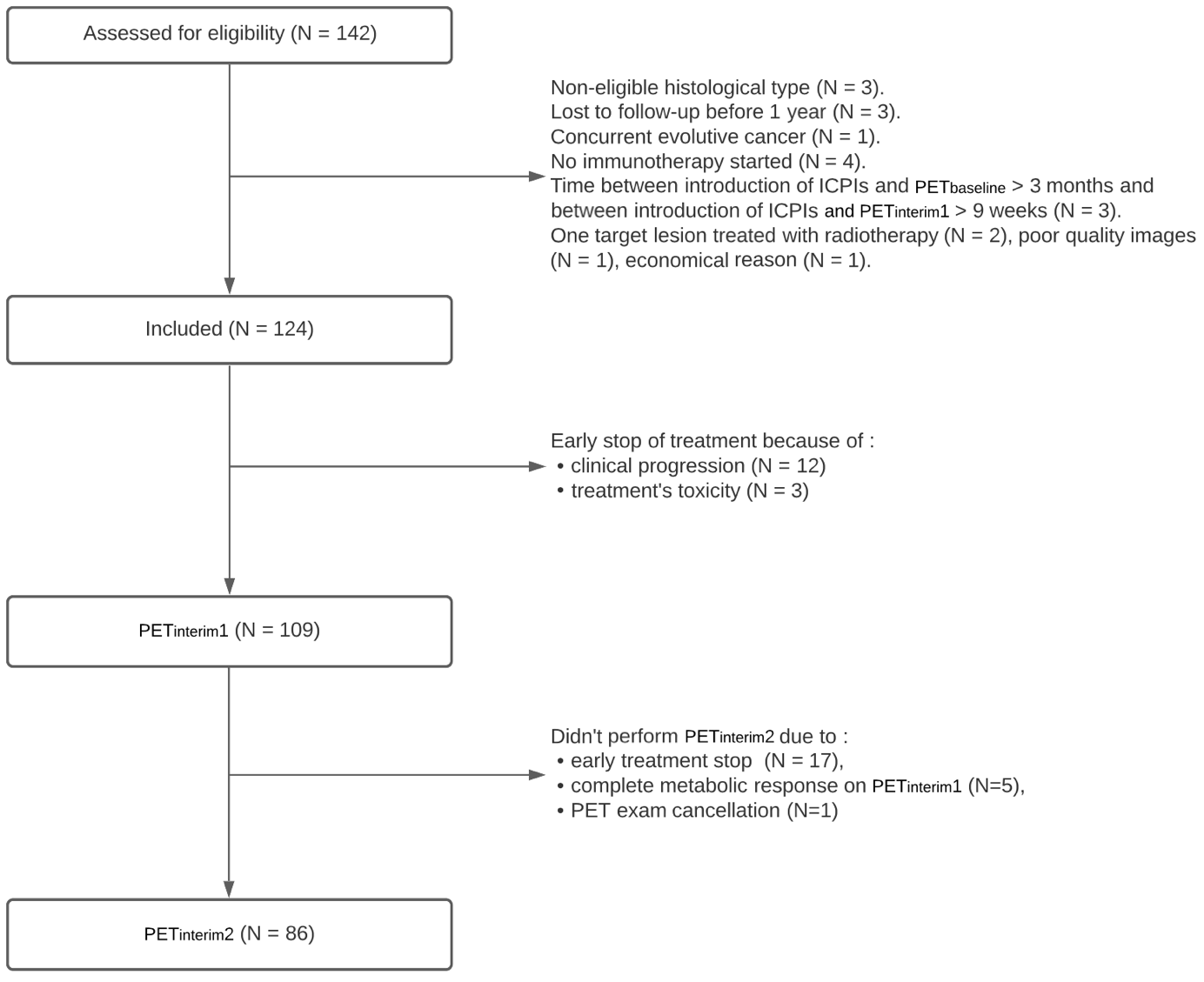
**Fig. S1**: Study flow-chart

| **PET_interim_1** | |
| --- | --- |
| Fig S2a. OS | 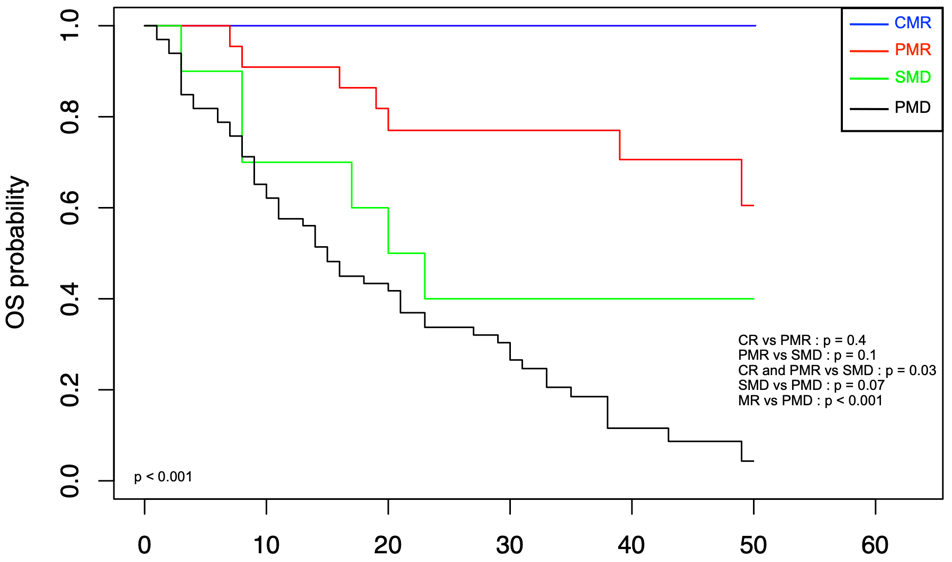  Time (months)  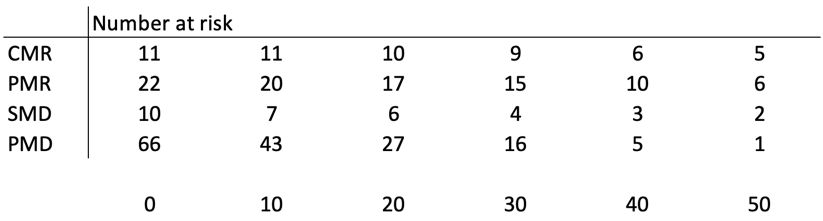  Time (months) |
| Fig S2b. PFS | 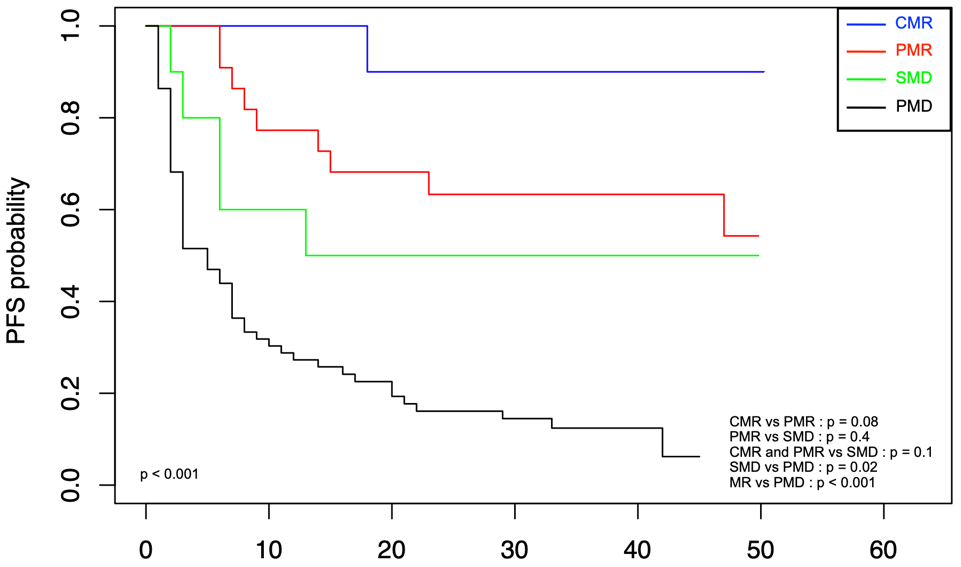  Time (months)  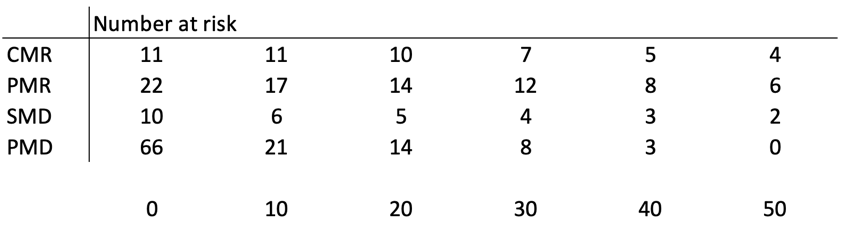  Time (months) |

**Figure S2a and S2b** : Kaplan-Meier survival curves showing overall survival (OS) (Fig. S2a) and progression free survival (PFS) (Fig. S2b) according to PERCIST tumor response on PET_interim_1. CMR: Complete Metabolic Response, PMR: Partial Metabolic Response, SMD: Stable Metabolic Disease, PMD: Progressive Metabolic Disease, MR: Metabolic Response.

| **PET_interim_2** | |
| --- | --- |
| Fig S3a. OS | 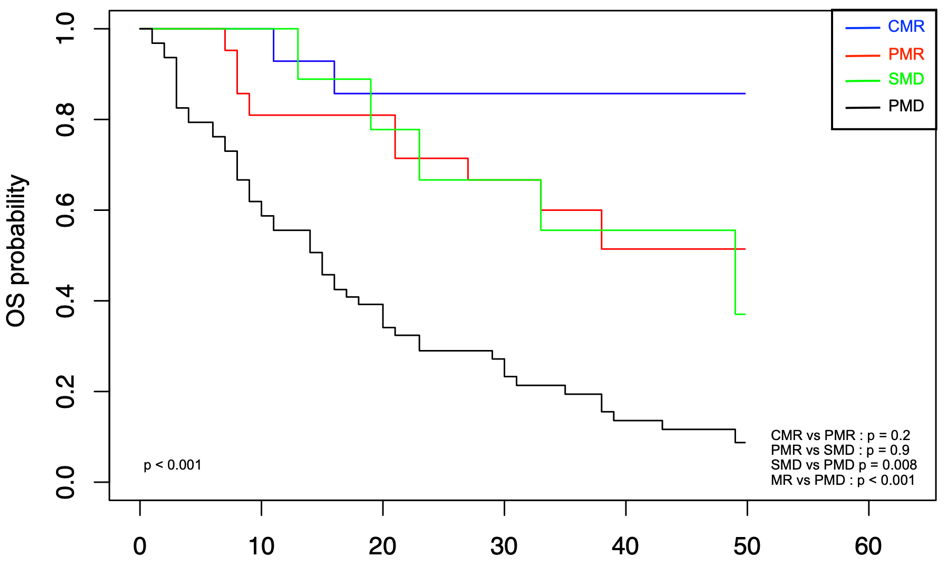  Time (months)  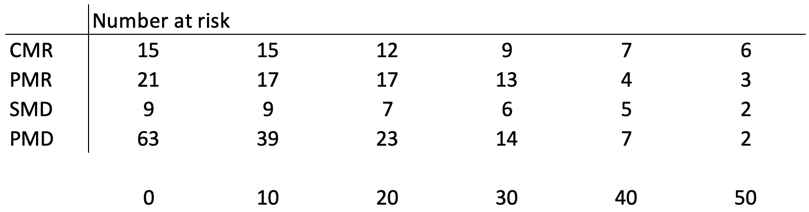  Time (months) |
| Fig S3b. PFS | 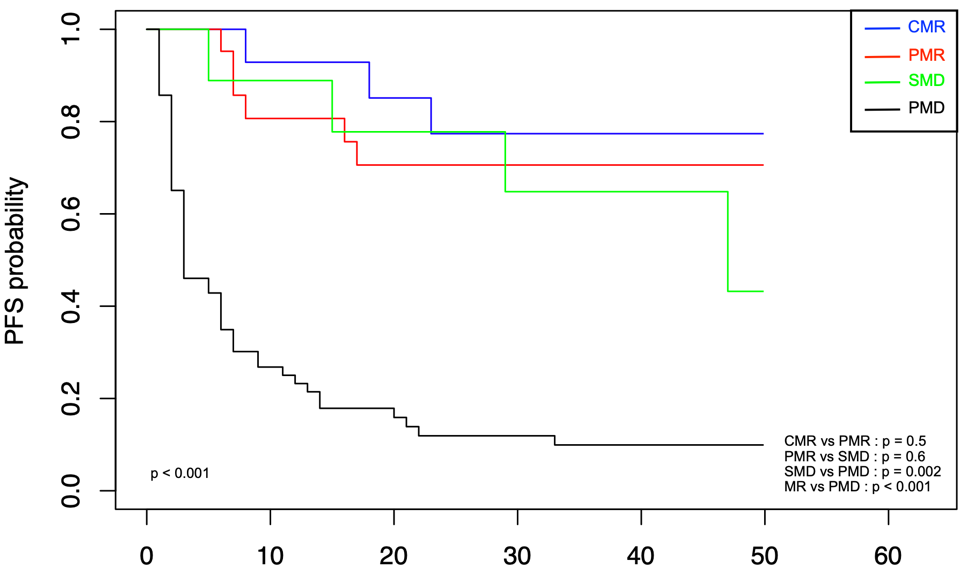  Time (months)  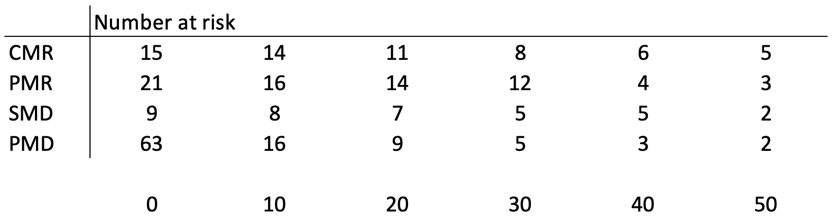  Time (months) |

**Figure S3a and S3b** : Kaplan-Meier survival curves showing overall survival (Fig. S3a) and progression free survival (Fig. S3b) according to PERCIST tumor response on PET_interim_2.

CMR: Complete Metabolic Response, PMR: Partial Metabolic Response, SMD: Stable Metabolic Disease, PMD: Progressive Metabolic Disease.


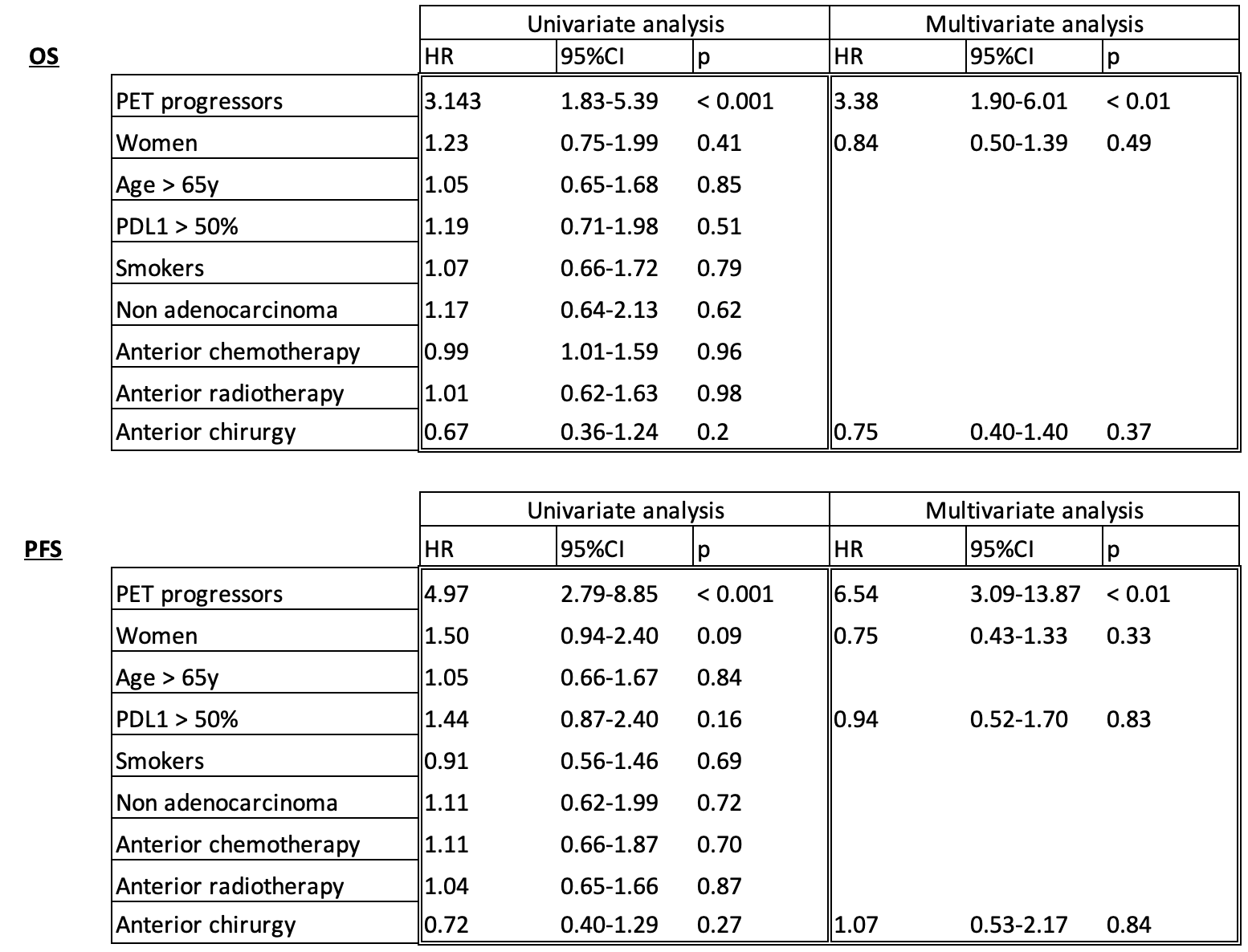


**Figure S4**: Factors associated with overall survival and progression free survival in the cox proportional hazard final model. HR = Hazard Ratio, 95%CI = 95% Confidence Interval.


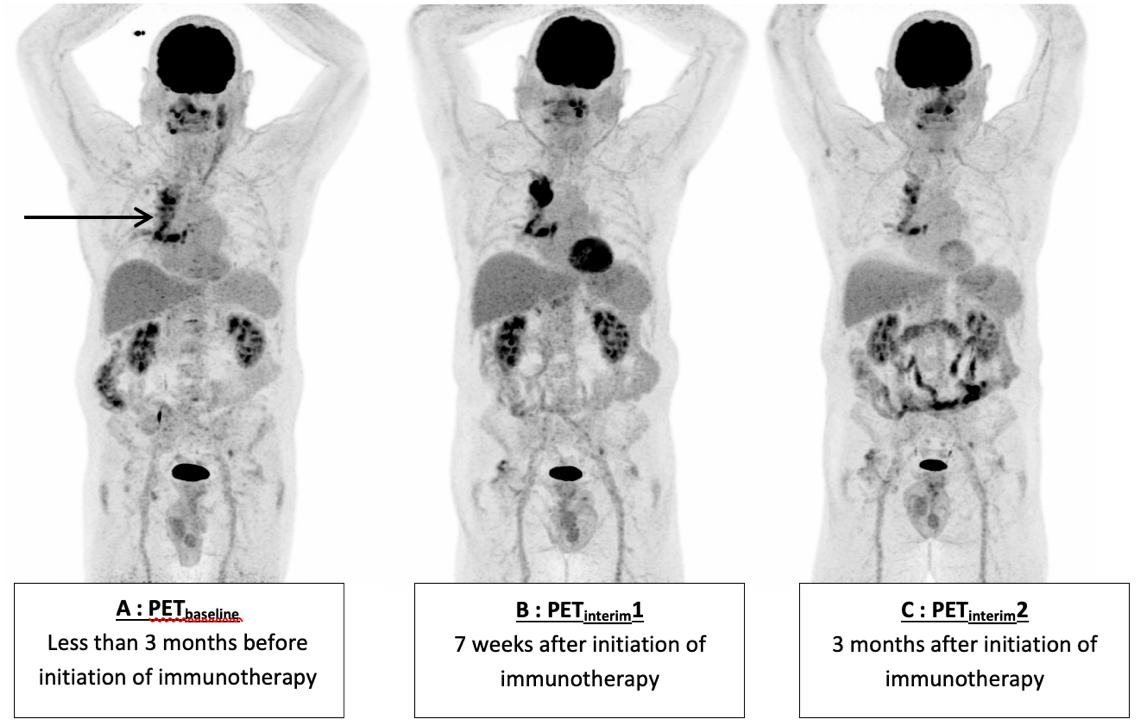


**Figure** **S5**: Metabolic progression 7 weeks after the introduction of immunotherapy (B) manifested by progression of the right upper lobar pulmonary lesion (continuous arrow) and stability of mediastinal-hilar nodal lesions. Then, at 3 months, partial response of all lesions indicating an initial pseudoprogression (C). This response was maintained at 17 months with an OS of 33 months.

| **PET_interim_1** | |
| --- | --- |
| Fig S6a. OS | 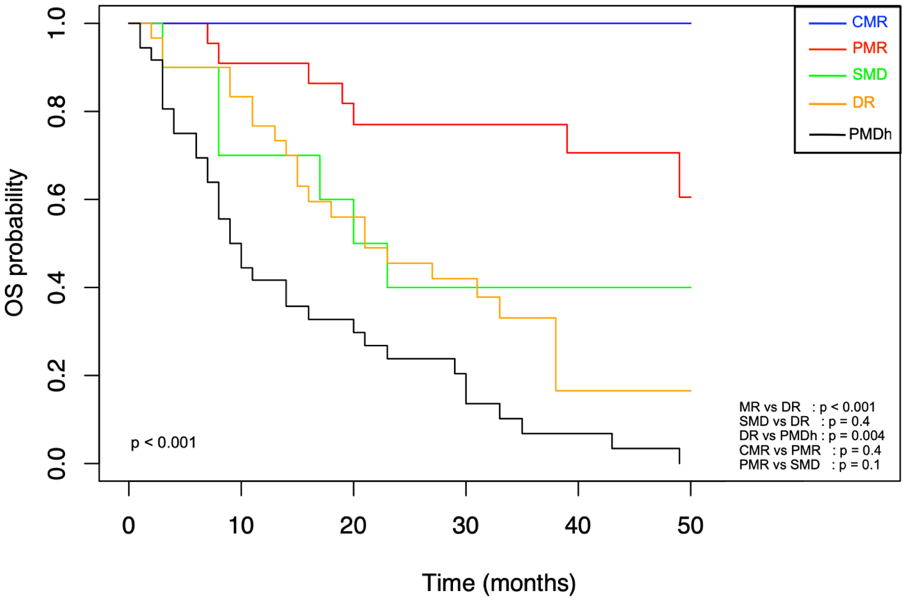  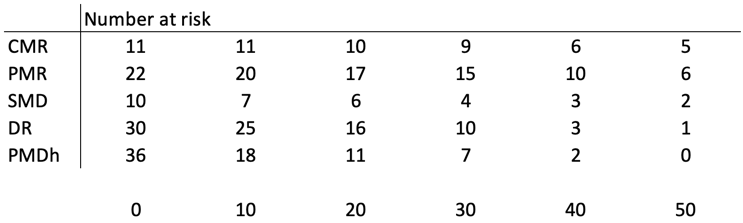  Time (months) |
| Fig S6b. PFS | 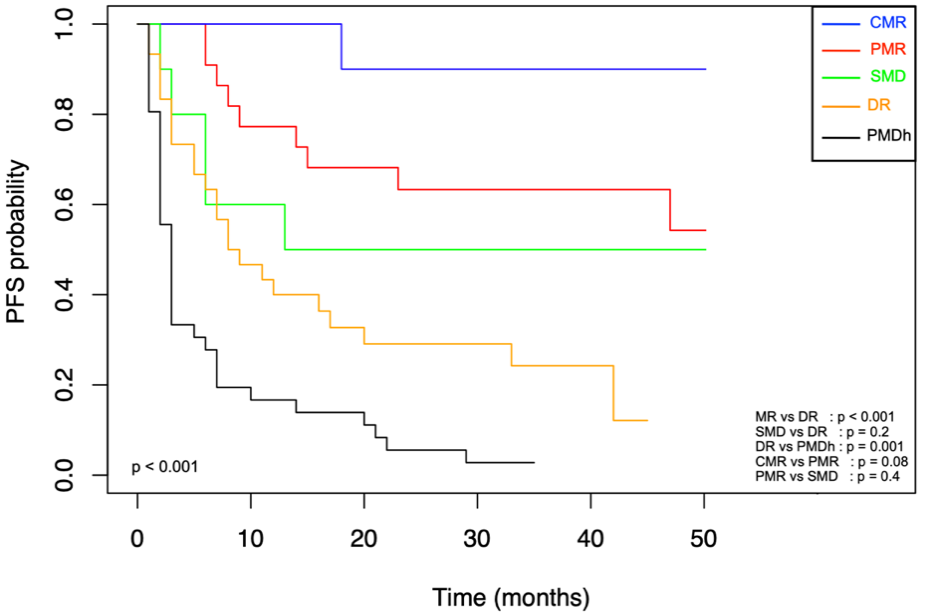  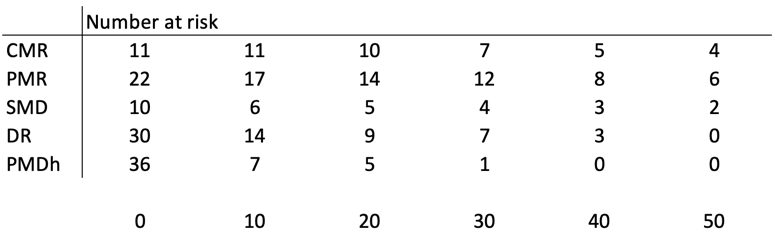  Time (months) |

**Figure S6a and S6b** : Kaplan-Meier survival curves showing overall survival (OS) (Fig. S6a) and progression free survival (PFS) (Fig. S6b) according to tumor response on PET_interim_1, including atypical response. CMR : Complete Metabolic Response, PMR : Partial Metabolic Response, SMD : Stable Metabolic Disease, DR : Dissociated Response, PMDh : homogeneous Progressive Metabolic Disease.


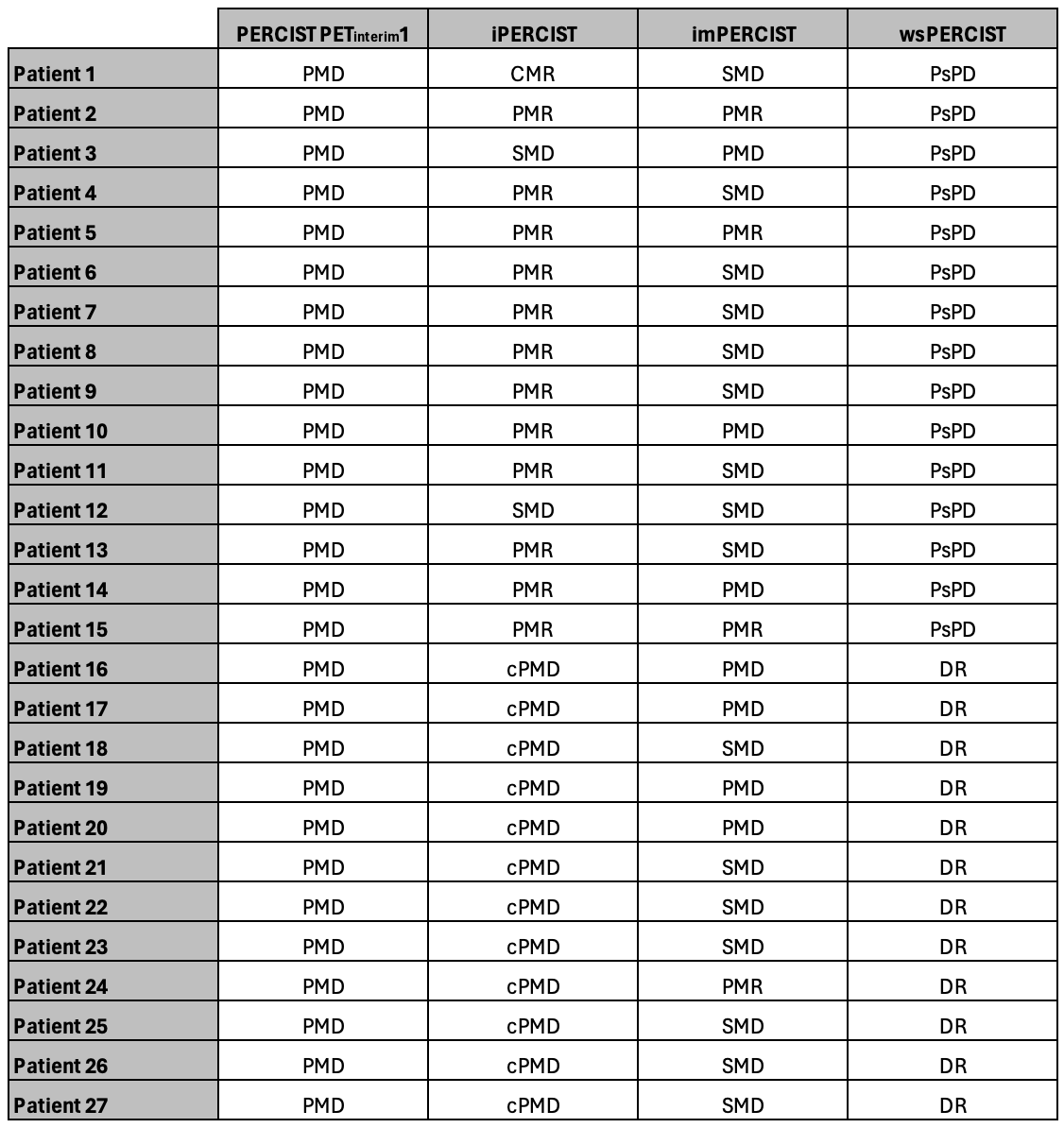


**Table S2**: Comparison of PERCIST, iPERCIST, imPERCIST and wsPERCIST criteria in the subgroups of patients with atypical responses (N=27).

PMD: Progressive Metabolic Disease; cPMD: confirmed Progressive Metabolic Disease; SMD: Stable Metabolic Disease; PMR: Partial Metabolic Response; PsPD: Pseudoprogression; DR: Dissociated Response

| Progressive metabolic disease (PMD) | Increase > 30% of the sum of SULpeak of target lesions from baseline scan or  visible increase in extent of tumor uptake or  new avid lesions typical of cancer. |
| --- | --- |
| Stable metabolic disease (SMD) | Not CMR, PMR, or PMD. |
| Partial Metabolic Response (PMR) | Reduction > 30% of the sum of SULpeak of target lesions and absolute drop in SUL > 0.8 SUL units. |
| Complete Metabolic Response (CMR) | Complete resolution of FDG uptake within measurable target lesion (less than mean liver activity and indistinguishable from surrounding background blood-pool levels).  No new lesion typical of cancer. |

**Table S1** : PERCIST 1.0 criteria

|  | **PERCIST PET_interim_1** | **iPERCIST** | **imPERCIST** | **wsPERCIST** |
| --- | --- | --- | --- | --- |
| **Patient 1** | PMD | CMR | SMD | PsPD |
| **Patient 2** | PMD | PMR | PMR | PsPD |
| **Patient 3** | PMD | SMD | PMD | PsPD |
| **Patient 4** | PMD | PMR | SMD | PsPD |
| **Patient 5** | PMD | PMR | PMR | PsPD |
| **Patient 6** | PMD | PMR | SMD | PsPD |
| **Patient 7** | PMD | PMR | SMD | PsPD |
| **Patient 8** | PMD | PMR | SMD | PsPD |
| **Patient 9** | PMD | PMR | SMD | PsPD |
| **Patient 10** | PMD | PMR | PMD | PsPD |
| **Patient 11** | PMD | PMR | SMD | PsPD |
| **Patient 12** | PMD | SMD | SMD | PsPD |
| **Patient 13** | PMD | PMR | SMD | PsPD |
| **Patient 14** | PMD | PMR | PMD | PsPD |
| **Patient 15** | PMD | PMR | PMR | PsPD |
| **Patient 16** | PMD | cPMD | PMD | DR |
| **Patient 17** | PMD | cPMD | PMD | DR |
| **Patient 18** | PMD | cPMD | SMD | DR |
| **Patient 19** | PMD | cPMD | PMD | DR |
| **Patient 20** | PMD | cPMD | PMD | DR |
| **Patient 21** | PMD | cPMD | SMD | DR |
| **Patient 22** | PMD | cPMD | SMD | DR |
| **Patient 23** | PMD | cPMD | SMD | DR |
| **Patient 24** | PMD | cPMD | PMR | DR |
| **Patient 25** | PMD | cPMD | SMD | DR |
| **Patient 26** | PMD | cPMD | SMD | DR |
| **Patient 27** | PMD | cPMD | SMD | DR |

**Table S2**: Comparison of PERCIST, iPERCIST, imPERCIST and wsPERCIST criteria in the subgroups of patients with atypical responses (N=27).

PMD: Progressive Metabolic Disease; cPMD: confirmed Progressive Metabolic Disease; SMD: Stable Metabolic Disease; PMR: Partial Metabolic Response; PsPD: Pseudoprogression; DR: Dissociated Response
